# Endovenous Laser Ablation of Varicose Veins Preserves Biological Properties of Vascular Endothelium and Modulates Proinflammatory Agent Profile More Favorably Than Classic Vein Stripping

**DOI:** 10.1155/2017/6167480

**Published:** 2017-02-20

**Authors:** Paweł Uruski, Krzysztof Aniukiewicz, Justyna Mikuła-Pietrasik, Patrycja Sosińska, Andrzej Tykarski, Krzysztof Książek, Zbigniew Krasiński

**Affiliations:** ^1^Department of Hypertensiology, Angiology and Internal Medicine, Poznań University of Medical Sciences, Długa 1/2 Str., 61-848 Poznań, Poland; ^2^Department of General and Vascular Surgery, Poznań University of Medical Sciences, Długa 1/2 Str., 61-848 Poznań, Poland; ^3^Department of Pathophysiology, Poznań University of Medical Sciences, Rokietnicka 8 Str., 60-806 Poznań, Poland

## Abstract

Here we compared effect of serum from varicose patients undergoing endovenous laser ablation (EVLA) and classic vein stripping (CVS) on biological properties of endothelial cells and on the local and systemic profiles of proinflammatory agents. Results showed that serum from EVLA patients improved proliferation and reduced senescence and oxidative stress in the endothelial cells, as compared with the serum from CVS patients. These effects were related to a suppressed activity of TGF-*β*1, the level of which in the serum from the EVLA patients was decreased. Medium generated by the cells subjected to EVLA serum contained decreased amounts of ICAM-1, VCAM-1, and E-selectin and increased amount of uPA, whereas the serum itself contained decreased concentrations of ICAM-1, E-selectin, and P-selectin and increased concentrations of uPA, PAI-1, and TFPI. Both EVLA and CVS resulted in diversified patients' reaction with respect to a direction of postprocedure changes in proinflammatory factors' serum level. Analysis of proportions showed that the groups differed remarkably in case of ICAM-1 and ET-1, the level of which declined in a higher fraction of patients treated endovenously. Our findings indicate that EVLA preserves better than CVS the functionality of vascular endothelium and modulates better both local and systemic profile of proinflammatory mediators.

## 1. Introduction

Varicose veins belong to the most frequent lifestyle diseases, as they affect up to 40% of industrialized countries' citizens in the age between 30 and 70 [1]. Etiology of the disease involves weakness of the vein wall and venous dilatation, elicited by abnormal venous wall remodelling [2]. Patients with varicosity suffer from multiple complications resulting from hemodynamic vein malfunction, such as skin discoloration, ulceration, thrombotic disorders, and hemorrhage [3].

Currently there are two ways for varicose vein management: lifestyle modifications and medical procedures. Lifestyle-related recommendations include the avoidance of a prolonged standing and sitting, an intensification of physical exercise, a loosening of restrictive clothes, and losing weight by obese people [4]. Medical methods include the use of venoactive drugs, compression treatment, sclerotherapy, phlebectomy, open venous surgery with ligation and stripping, and endovenous ablation techniques [3, 5].

Until the past few years, classic surgical methods of varicose vein removal—mainly vein stripping—were considered as the most radical and effective ways to cope with the pathology. On the other hand, traumatizing nature of these methods yielded several side effects [5] which directed surgeons' attention to less invasive treatment modalities, in particular endovenous laser ablation (EVLA). Comparative studies in which the outcomes of patients undergoing classic vein stripping (CVS) and EVLA were analyzed revealed that the latter experienced lower postoperative pain and bleeding, decreased frequency of wound infections and hematoma, and shorter time of convalescence [6, 7]. Literature is, however, not fully consistent in this regard, as there are also reports in which some postintervention effects of both kinds of surgery (e.g., pain, thrombosis, and bleeding) were comparable [7, 8].

In this paper we joined the debate and compared patients treated with CVS and EVLA in terms of an effect of their serum on such critical, functional features of endothelial cells, like proliferation, senescence, production of reactive oxygen species (ROS), and secretory properties. These in vitro investigations were followed by a comparative ex vivo analysis of both groups of sera with respect to a level of eight arbitrarily selected mediators of vascular inflammation. Last but not least, the postsurgery changes in the serum concentration of tested mediators were evaluated.

## 2. Materials and Methods

### 2.1. Materials

Unless otherwise stated, all chemicals and culture plastics were purchased from Sigma (St. Louis, MO). Exogenous, recombinant forms of human GRO-1 and TGF-*β*1 were obtained from R&D Systems (Abingdon, UK).

### 2.2. Surgical Procedures

The study included 40 patients undergoing surgical treatment for primary varicose veins. All subjects had symptomatic disease and the inclusion criteria for the study were based on clinical examination. Half of patients received CVS and the second half was treated with EVLA. Both procedures were performed under spinal anesthesia. The exclusion criteria included ankle brachial pressure index (ABPI) < 0.8, previous deep vein thrombosis or pulmonary embolism, coagulopathy, malignancy, and pregnancy. Demographic data regarding patients are presented in Table 1.

During CVS, two incisions were made: one at the groin and one at the ankle. The saphenous vein was then opened at each end and a flexible wire was inserted into the entire vein and then pulled out to remove the vein. During EVLA, an access to the distal part of the vein was achieved through percutaneous puncture under ultrasound control. The working device (810 nm wavelength axial diode laser; EVLA810) was introduced and advanced towards the saphenofemoral junction. After confirmation of the position of the tip of the working device (1-2 cm from the saphenofemoral junction) in ultrasonography, the ablation was performed with the aim to deliver 60–100 J for 1 cm of the treated vein. The study was approved by the institutional ethics committee (consent number 441/13).

### 2.3. Serum Samples

The samples of serum were collected from patients undergoing surgery due to varicose veins of lower extremities 1 h before surgery (point A) and 6 h after surgery (point B). Blood was centrifuged immediately after collection and serum samples were stored in aliquots at −80°C until required.

### 2.4. Endothelial Cell Culture

Human umbilical vein endothelial cells (HUVECs) were purchased in the ATCC (Rockville, MD, USA). The cells were cultured in DMEM with 20% FBS, L-glutamine (2 mM), HEPES (20 mM), EGF (10 *μ*g/mL), heparin (5 U/mL), penicillin (100 U/mL), and streptomycin (100 *μ*g/mL). Upon reaching confluency, cells were exposed to 20% serum (point B) for 72 h. Then the cells were carefully washed with phosphate buffered saline (PBS) and exposed to serum-free medium for 72 h to generate autologous medium.

### 2.5. Immunoassays

Concentration of proinflammatory agents was measured using appropriate DuoSet® Immunoassay Development kits (R&D Systems), according to manufacturer's instructions.

### 2.6. Determination of Endothelial Cell Proliferation and Senescence

Endothelial cells were seeded into culture dishes at low density (5 × 10^4^ cells per well), allowed to attach for 2 h, and then growth synchronized by serum deprivation for 4 h. Afterwards, the cells were exposed to 20% serum obtained from patients undergoing CVS and EVLA (point B) for 72 h. After the incubation, cells were detached and counted, and the number of population doublings (the measure of cell proliferation) was calculated using the following formula: population doublings = log_2_⁡(*C*_*t*_/*C*_*o*_), where *C*_*o*_ indicates the number of cells inoculated and *C*_*t*_ indicates the number of cells harvested.

The activity of senescence-associated *β*-galactosidase (SA-*β*-Gal) was quantified by measuring the rate of conversion of 4-methylumbelliferyl-*β*-D-galactopyranose to 4-methylumbelliferone at pH 6.0, essentially as described in [9]. In some experiments, cell proliferation and activity of SA-*β*-Gal were monitored upon cell exposure to exogenous, recombinant forms of GRO-1 and TGF-*β*1.

### 2.7. Measurement of Reactive Oxygen Species (ROS)

ROS production was assessed in endothelial cells exposed to 20% serum (point B) from patients treated with CVS and EVLA for 72 h. In brief, 1 × 10^5^ cells were incubated in the presence of 5 *μ*M 2′,7′-dichlorodihydrofluorescein diacetate (H_2_DCFDA) (Molecular Probes, Eugene, USA) for 45 min at 37°C. The fluorescence intensity in cell lysates was monitored in a spectrofluorometer Victor2 (Perkin-Elmer, Turku, Finland) with excitation at 485 nm and emission at 535 nm. In some experiments, the production of ROS was examined upon cell exposure to exogenous GRO-1 and TGF-*β*1.

### 2.8. Statistics

Statistical analysis was performed using GraphPad Prism™ v.5.00 software (GraphPad Software, San Diego, USA). The means were compared with Mann–Whitney* U* test or Wilcoxon signed-rank test, when appropriate. The differences between proportions were analyzed using Fisher's exact test using Analyse-It 1.71 software (Analyse-It Software, Leeds, UK). Results are expressed as means ± SEM. Differences with a *P* value < 0.05 were considered to be statistically significant.

## 3. Results

### 3.1. Functional Properties of Vascular Endothelium Are Better Preserved in the Presence of Serum from Patients Treated with EVLA Than with CVS

**Figure 1:**
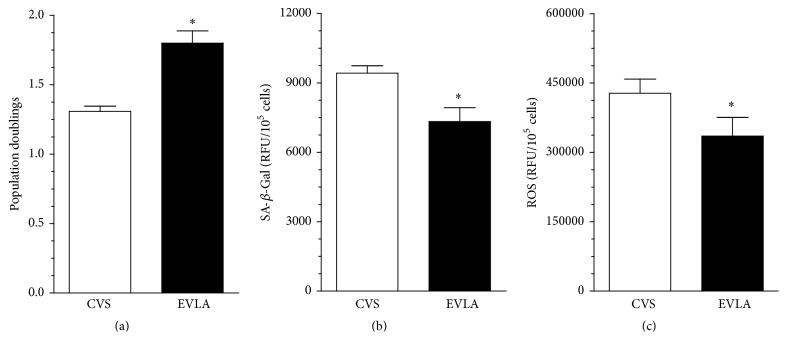
Effect of serum from patients treated with CVS and EVLA on the number of population doublings (a), the activity of SA-*β*-Gal (b), and the production of reactive oxygen species (c) in endothelial cells. Endothelial cells were exposed to 20% sera for 72 h. Results (expressed as means ± SEM) derive from experiments performed with the sera from 8 (proliferation) or 12 (senescence, oxidative stress) patients per group. Asterisks indicate significant differences as compared with CVS. RFU: relative fluorescence units.

Low-density HUVECs were exposed to 20% serum from patients treated with CVS and EVLA for 72 h and then three parameters being the measures of cellular stress, that is, cell proliferation, activity of SA-*β*-Gal (a marker of senescence), and the generation of ROS (an indicator of oxidative stress), were investigated. The experiments showed that the cells subjected to sera from patients treated with EVLA displayed increased number of population doublings achieved, decreased activity of SA-*β*-Gal, and decreased production of ROS, as compared with the cells exposed to sera from CVS patients (Figure 1).

### 3.2. Improved Functionality of Endothelial Cells Exposed to Serum from Patients Undergoing EVLA Is Associated with Suppressed Activity of TGF-*β*1

**Figure 2:**
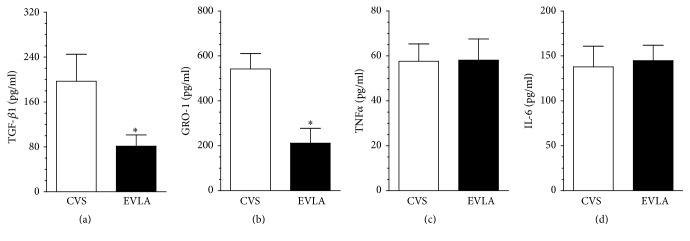
Concentration of TGF-*β*1 (a), GRO-1 (b), TNF*α* (c), and IL-6 (d) in the serum from patients treated with the classic vein stripping (CVS) and the endovenous laser ablation (EVLA). The measurements were made using the appropriate ELISA kits using serum collected 6 h after surgery. Results (expressed as means ± SEM) derive from experiments performed with the sera from 7 (TGF-*β*1, GRO-1), 10 (TNF*α*), and 12 (IL-6) patients per group. Asterisks indicate significant differences as compared with CVS.

Both groups of sera were compared with respect to a concentration of four arbitrarily selected cytokines (TGF-*β*1, GRO-1, TNF*α*, and IL-6), the activity of which has already been linked with the inhibition of cell growth [10] and the promotion of cellular senescence [11, 12] and oxidative stress [13, 14]. Study showed that serum from the EVLA patients contains lower concentration of TGF-*β*1 and GRO-1 than serum from the CVS patients. At the same time, concentration of TNF*α* and IL-6 in both groups of sera was comparable (Figure 2).

**Figure 3:**
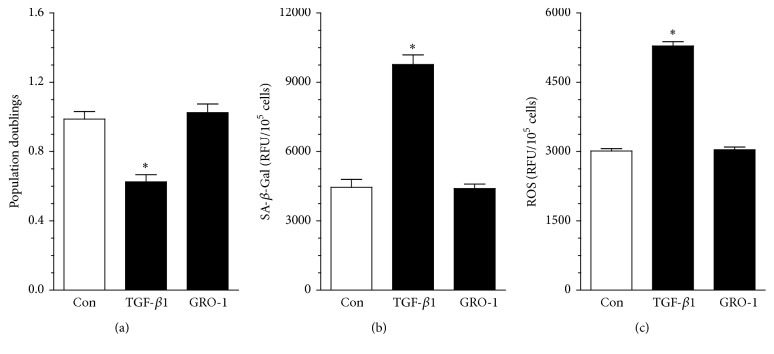
Effect of exogenous, recombinant TGF-*β*1 and GRO-1 on proliferation (a), senescence (b), and oxidative stress (c) in endothelial cells. The cells were subjected to exogenous proteins used at the doses corresponding to their serum (for CVS patients) levels for 72 h. Results (expressed as means ± SEM) derive from experiments performed in octuplicate. Asterisks indicate significant differences as compared with the control group (cells maintained in the standard conditions). RFU: relative fluorescence units.

In order to verify if TGF-*β*1 and/or GRO-1 could be responsible for the dysfunction of cells exposed to serum from CVS patients, recombinant forms of these proteins were applied to HUVECs in the concentrations corresponding to the average serum level of these cytokines (200 pg/mL and 500 pg/mL for TGF-*β*1 and GRO-1, respectively), and then three abovementioned aspects of endothelial cell biology were reexamined. Experiments showed that exogenous TGF-*β*1 is capable of inhibiting proliferation, as well as inducing SA-*β*-Gal and ROS in HUVECs, whereas exogenous GRO-1 failed to affect these phenomena (Figure 3).

### 3.3. Serum from EVLA Patients Generates More Favorable Pattern of Proinflammatory Agents in the Culture and Systemic Conditions Than Serum from CVS Patients

**Figure 4:**
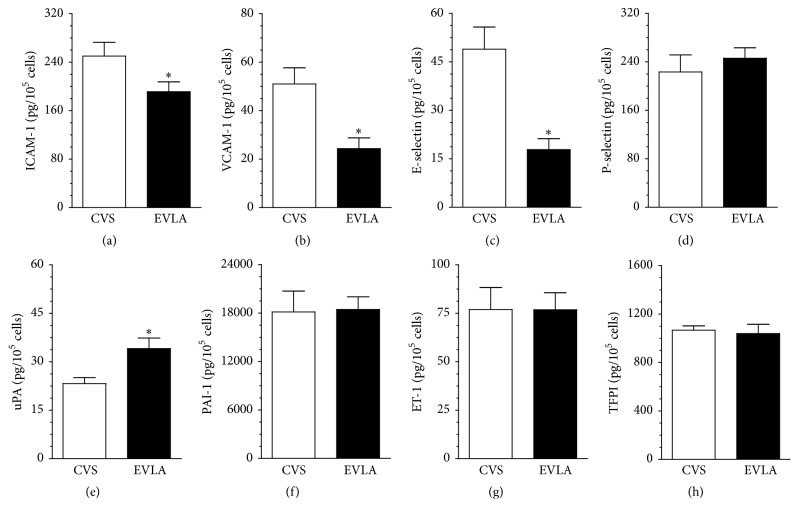
Concentration of proinflammatory agents in conditioned medium generated by endothelial cells subjected to serum from patients with varicose veins treated with classic vein stripping (CVS) and endovenous laser ablation (EVLA). Endothelial cells were subjected to 20% serum collected 6 h after surgery for 72 h and then they were washed and exposed for the next 72 h to serum-free medium to generate conditioned medium in which proinflammatory agents were quantified. Results (expressed as means ± SEM) derive from experiments performed with the sera from 12 patients per group. Asterisks indicate significant differences as compared with CVS.

Conditioned medium from HUVECs exposed to sera (point B) from the EVLA patients contained decreased concentration of three out of eight investigated mediators of vascular inflammation, that is, ICAM-1, VCAM-1, and E-selectin, as compared with the cells exposed to sera from the CVS patients. In addition, these media contained increased level of uPA. Concentrations of the remaining agents (P-selectin, PAI-1, ET-1, and TFPI) in both groups of sera were comparable (Figure 4).

**Figure 5:**
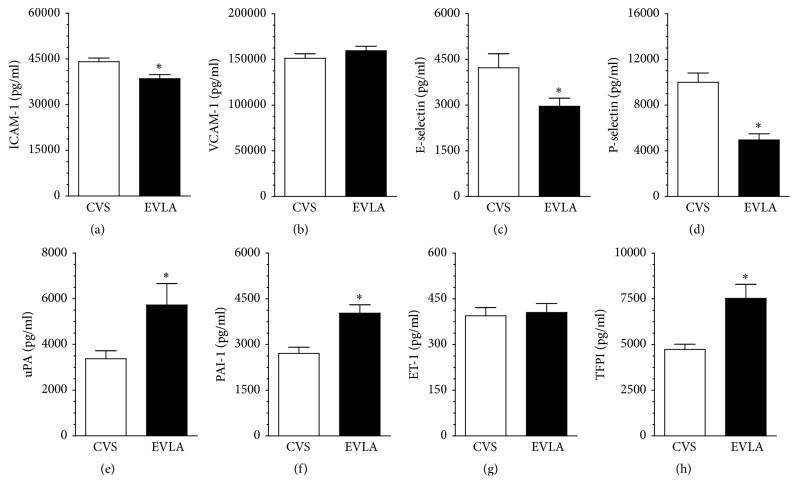
Concentration of proinflammatory agents in serum from the patients with varicose veins treated with the classic vein stripping (CVS) and the endovenous laser ablation (EVLA).The measurements were made using the appropriate ELISA kits using the sera collected 6 h after surgery. Results (expressed as means ± SEM) derive from the analysis of sera obtained from 20 patients per group. Asterisks indicate significant differences as compared with CVS.

Ex vivo studies in which concentration of the same molecules was measured directly in the sera obtained from both groups of varicose patient showed that the samples from EVLA patients contained lower concentration of ICAM-1, E-selectin, and P-selectin and higher concentration of uPA, PAI-1, and TFPI, as compared with the serum from CVS patients. Concentration of two remaining agents, that is, VCAM-1 and ET-1, in both groups was similar (Figure 5).

### 3.4. EVLA Outcome Is Less Proinflammatory as Compared with CVS

In order to analyze results of CVS and EVLA in terms of changes in the concentration of tested proinflammatory agents, a level of a given agent in the serum obtained after surgery (point B) was compared with its level before the procedure (point A). This study revealed that in case of every mediator tested there is a group of patients in whom both types of surgery lead to an increase in certain agents' level and a second group of patients in whom the level of those agents declines.

This heterogenous reaction prompted us to divide the patients into two groups (marked as “decreases” and “increases”) and to make some assumptions regarding desired changes. Namely, we assumed that if EVLA is truly less proinflammatory than CVS, it should lead to the decreases in the level of tested agents in a higher percentage of patients. Per analogy, any increases in the level of the tested agents upon EVLA should occur in a lower fraction of patients, as compared with CVS. Analysis performed using such an algorithm followed by a proportion analysis with Fisher's exact test showed that abovementioned assumptions were met in case of two agents, that is, ICAM-1 and ET-1. For the rest of studied mediators, a surgery-related changes in their serum level had no statistical significance (Table 2).

## 4. Discussion

Although the presence of varicose veins predisposes to the development of certain complications, medical treatment of the disease with a classic surgery (CVS) or new endovascular techniques (EVLA) may also result in various procedure-related morbidities [5]. At the same time, pathogenesis of only a few surgery-related complications, for example, pain, bleeding, and bruising, has mechanistically been explained and linked with specific features of a given procedure, including the degree of mechanical tissue traumatization, a technique of stripping/ligation, a catheter positioning, a type of laser apparatus, and the use of additional drugs [3, 5, 7, 15]. Interestingly, in case of complications, in which dysfunctional endothelium and local or systemic inflammation play a role (e.g., thrombotic disorders [3]), it is even not entirely clear which procedure, CVT or EVLA, is safer. One thing, which is sure, is that the tendency that can be observed in surgical units recently is the decreasing incidence of some EVLA complications (e.g., deep vein thrombosis [16, 17]), which likely reflects both the rising experience of surgeons in using this technique [18] and technological improvements in surgical devises [19]. These observations may lead to a hypothesis that EVLA has great potential to be less traumatic to cells and tissues than classic techniques. Unfortunately, the experimental evidences supporting this statement are very few.

Based on this we designed a project to compare the local (endothelium-related) and systemic (serum-related) outcomes of CVS and EVLA. Experiments showed that EVLA is less stressful for endothelial cells than CVS, as the serum obtained from patients undergoing this procedure fueled their proliferation and reduced senescence and oxidative stress. This improved functionality of endothelial cells upon EVLA, in particular decreased senescence, is of special importance with respect to the spreading of the pathology within normal vessels, of which conclusion stems from the histopathological analysis by Aunapuu and Arend showing that endothelial cells from varicose patients display specific discontinuity and denudation that may be a manifestation of cellular senescence [20].

Comparative analysis of sera from the CVS and EVLA groups showed that the former contained increased level of TGF-*β*1 and GRO-1, the cytokines with confirmed antiproliferative, and/or prosenescence capabilities [11, 12]. Further experiments with recombinant forms of these proteins revealed that only TGF-*β*1 was capable of inhibiting growth, as well as inducing senescence and oxidative stress in endothelial cells, which is in line with findings by other groups [10, 21].

Another indicator of endothelial cell dysfunction that was tested in the project was the cells' ability to secrete eight arbitrarily selected mediators of vascular inflammation, that is, adhesion molecules (ICAM-1, VCAM-1, E-selectin, and P-selectin), mediators of coagulation and fibrinolysis (uPA, PAI-1, and TFPI), and a vasoconstrictive agent, ET-1. Interestingly, cells exposed from serum from the EVLA patients adopted clearly less proinflammatory phenotype, as they secreted decreased amounts of ICAM-1, VCAM-1, and E-selectin. This finding implying that EVLA is, indeed, less traumatizing to endothelial cells than CVS agrees with other reports, for example, those describing that endothelium experiencing stressful conditions (elevated shear stress or expanded application of carbon nanotubes) exhibits increased expression of ICAM-1 and VCAM-1 [22, 23]. At the same time, HUVECs subjected to the serum from EVLA patients produced more uPA, which should be also considered as a beneficial effect, as this molecule belongs to fibrinolytic agents and thus may reduce the risk of thrombotic complications of the surgery [24].

Having established that EVLA exerts more favorable effects than CVS in local, cell-related conditions, we went to analyze the differences in the serum level of the same proinflammatory molecules that has been tested in vitro. Also in this case, EVLA suppressed the inflammatory serum phenotype, as the samples from patients treated with this procedure contained lower levels of ICAM-1, E-selectin, and P-selectin. The similarity of effects observed on cellular (conditioned medium) and systemic (serum) levels with respect to ICAM-1 and E-selectin may suggest that reduced serum content of these agents may directly result from their downregulated secretion by vascular endothelium. Taking into account the fact that both these molecules are typically shed to environment from the surface of activated endothelial cells [25], our findings indicate that pathological cell activation in EVLA patients is markedly lower. Interestingly, serum from EVLA patients also contained increased amounts of uPA, PAI-1, and TFPI, which indicates that the procedure also suppresses the thrombogenic aspect of the inflammatory reactions.

As per a direct outcome of a surgery (point B versus point A), the results obtained appeared to be very intriguing, as they showed a diversified patients' reaction. In fact, both the decreases and the increases in proinflammatory agent level upon both procedures were observed, confirming findings by Nesbitt et al. about high variability of varicose patient responses to a surgery [26]. In order to interpret these data properly, we have constructed an algorithm with which we found that EVLA decreases the serum levels of ICAM-1 and ET-1 in remarkably higher fraction of patients than CVS, confirming our primary hypothesis that biological effects of this technique are more favorable as compared with CVS. In this context, it should be stressed, however, that to some extent the results we obtained using EVLA with 810 nm laser may not be directly transferable to slightly more modern endovenous technique using a 1470 nm laser.

## 5. Conclusions

Collectively, our findings showing that EVLA is less traumatizing to vascular endothelium and generates lower level of proinflammatory mediators than CVS may suggest that cellular and systemic changes resulting from this procedure are safer for varicose patients from the perspective of the development of further endothelium- and inflammation-dependent morbidities. At the same time, it should be emphasized that that our findings point on the differences between these two procedures at the cellular level. To a significant extent, different outcomes of CVS and EVLA are also associated with different degree of tissue destruction. During the stripping the entire vein is removed, side branches are disrupted, and bleeding occurs into the stripping channel. During the laser technique, the vessel is left in place and damaged by heat from inside without concomitant injury of the surrounding tissue and bleeding. For this reason, either factors associated with altered cell functionality or technique characteristics should be taken into account during the final selection of a method of varicose vein surgery.

## Figures and Tables

**Table 1:** Characteristics of patients undergoing surgical varicose vein removal using CVS and EVLA.

Parameter	CVS	EVLA
*n*	20	20
Sex (male/female; *n*)	9/11	6/14
Age (mean ± SD/range; *y*)	56 ± 13/29–81	48 ± 16/21–78
	Comorbidities (number)	
No comorbidities	10	8
Hypertension	3	4
Diabetes mellitus	1	2
Hypothyroidism	2	1
Asthma	1	1
Obesity	0	2
Psoriasis	1	2
Allergy	1	0
Adrenal insufficiency	1	0

**Table 2:** Surgery-related changes in serum concentration of proinflammatory agents.

Agent	Decreases				Increases				*P* < 0.05
	CVS		EVLA		CVS		EVLA		
	*n*/total	%	*n*/total	%	*n*/total	%	*n*/total	%	
ICAM-1	9/20	45	17/20	85	11/20	55	3/20	15	Yes
VCAM-1	11/19	58	5/19	26	8/19	42	14/19	74	No
E-selectin	14/19	74	13/20	65	5/19	26	7/20	35	No
P-selectin	11/20	55	9/19	47	9/20	45	10/19	53	No
uPA	14/19	74	13/20	65	5/19	26	7/20	35	No
PAI-1	8/19	42	14/20	70	11/19	58	6/20	30	No
ET-1	8/20	40	15/20	75	12/20	60	5/20	25	Yes
TFPI	13/19	68	9/19	47	6/19	32	10/19	53	No

*n* value indicates the number of patients in whom the level of a given factor decreased or increased. *P* values refer to the made assumptions (see Results for details) and are determined by the statistical analysis of differences between proportions.
